# Setting health research priorities using the CHNRI method: III. Involving stakeholders

**DOI:** 10.7189/jogh.06.010303

**Published:** 2016-06

**Authors:** Sachiyo Yoshida, Kerri Wazny, Simon Cousens, Kit Yee Chan

**Affiliations:** 1 Department for Maternal, Newborn, Child and Adolescent Health, World Health Organization, Geneva, Switzerland; 2 Centre for Global Health Research, The Usher Institute for Population Health Sciences and Informatics, The University of Edinburgh, Edinburgh, Scotland, UK; 3 Department of Infectious Disease Epidemiology, London School of Hygiene and Tropical Medicine, London, UK; 4 Nossal Institute for Global Health, University of Melbourne, Melbourne, Australia; * These authors contributed equally to the work.

Setting health research priorities is a complex and value–driven process. The introduction of the Child Health and Nutrition Research Initiative (CHNRI) method has made the process of setting research priorities more transparent and inclusive, but much of the process remains in the hands of funders and researchers, as described in the previous two papers in this series [1,2]. However, the value systems of numerous other important stakeholders, particularly those on the receiving end of health research products, are very rarely addressed in any process of priority setting. Inclusion of a larger and more diverse group of stakeholders in the process would result in a better reflection of the system of values of the broader community, resulting in recommendations that are more legitimate and acceptable.

The CHNRI method, as originally proposed, took into account the importance of stakeholders and made provisions for their participation in the process. Although the involvement of a large and diverse group of stakeholders is desirable, they were not expected to propose research ideas, or score them against the set of pre–defined criteria. Because of this, the original CHNRI method proposed that stakeholders should be allowed to “weigh” pre–defined criteria and set “thresholds” for a minimum acceptable score against each criterion that would be required for a research idea to be considered a “research priority”. In choosing the stakeholders, the context of each exercise will be very important and the goals of the specific exercise should be defined before choosing an appropriate “stakeholder group”. Among stakeholders, we would expect to see those affected by the disease of interest and their family members, their carers and health workers, members of general public, media representatives interested in the topic, community leaders, representatives of the consumer groups and industry, but also potentially researchers and funders themselves. Although the latter two groups – researchers and funders – already have a different role assigned in the CHNRI process, this does not exclude them from also being stakeholders in the process [1,2]. In this paper, we aim to review and analyse the experiences in stakeholder involvement across the 50 CHNRI exercises published in the 10–year period between 2007 and 2016, the proposed approaches to involving stakeholders and their effects on the outcome of the prioritization process.

One paper in the original CHNRI method series focused on involving stakeholders [3]. That paper presented practical experiences from three separate attempts to involve stakeholders that took place in 2006. The three groups approached were: (i) members of the global research priority setting network; (ii) a diverse group of national–level stakeholders from South Africa; and (iii) participants at a conference related to international child health held in Washington, DC, USA. Each group was asked to complete a short questionnaire to assess the relative importance of the five original CHNRI criteria. Different versions of the questionnaire were used with each group [3]. The results of this exercise indicated that groups of stakeholders vary in the weights they assigned to the 5 criteria, reflecting divergence in the “value” placed on each criterion by each stakeholder group.

The diverse group of respondents within the priority–setting network placed the greatest weight on the criterion of “maximum potential for disease burden reduction” and the most stringent threshold on “answerability in an ethical way”. Among the attendees at the international conference on child health, the criterion of “deliverability, answerability and sustainability” was identified as the most important. Finally, in South Africa, where inequity has been a national problem historically, the greatest weight was placed on the “predicted impact on equity” criterion.

This comparative analysis by Kapiriri et al. [3] effectively demonstrated that involving a wide range of stakeholders is an important goal for any research priority setting exercise. The criteria that may be of importance to funders, scientists and other technical experts involved in the process of planning and conducting the exercise may not be well aligned with the values of those who should eventually benefit from health research, or with the sentiments of wider society as a whole [3]. This is an important observation, because if the CHNRI process is conducted without regard for the broader social value or research, then it is unrealistic to expect it to fulfil its purpose of being accepted as a fair, transparent and legitimate process for setting investment priorities for health research.

## THE CONCEPTS OF THRESHOLDS AND WEIGHTS IN THE CHNRI METHOD

These concepts were introduced as a part of the initial CHNRI method description [4,5]. The multi–disciplinary working group that developed the CHNRI method recognised the need to find a practical way to involve a much larger group of stakeholders in the priority–setting process. An agreement was reached that, at least in principle, most members of the public would not be expected to generate research ideas or score them, because they do not possess the knowledge that would enable them to discriminate among the proposed research ideas. Instead, it was agreed that their contribution to the process and the final results of the exercise would be be in the assignment of “weights” to the criteria that reflect their collective preferences and beliefs. Over the years of CHNRI implementation, it has been shown that stakeholders originating from funding institutions or political organizations prefer the criterion of maximum potential for disease burden reduction, because their targets are usually set around this criterion; programme managers are typically more focused on the deliverability and sustainability criterion; stakeholders from the industry tend to prefer knowing the likelihood of effectiveness of resulting interventions; while members of the general public often emphasize equity and ethics as their preferred criteria [6].

In addition to placing more “weight” on some criteria than others, which could affect the final rankings of *all* research ideas as a result of stakeholders' input into the CHNRI process, the stakeholders can also disqualify *some* research ideas using the system of “thresholds”. This means they may agree a priori that a research idea will not be considered a priority unless it reaches a certain minimum score against a particular priority–setting criterion. This can be important in a specific context; eg, in the aforementioned example of South Africa, where equity was a very important concern for all stakeholders, they could have insisted that a research idea must have a minimum score of 80% on the “equity” criterion to qualify as a priority. In practice, this means that a research idea with scores 50–70% on all other criteria, but 90% on “equity”, could be considered a research priority. However, another idea with scores of 80–90% on all other criteria, but 60% on “equity” would be disqualified from the exercise – or at least delayed, until it addresses the recognized issues with equity. Common examples of the latter are the new, high technology–based interventions that would likely first be utilised by the wealthy. In this way, research ideas with lower overall scores could be seen as greater priorities if they pass all the pre–defined “thresholds” [3,4].

Although the interdisciplinary group that developed the CHNRI method considered this approach as practical and inclusive, the question remained of how best to select the stakeholders and ensure their representativeness to the entire community of interest. Possibly the best solution to this problem to date has been achieved by Kapiriri et al. [3] who aimed to develop a “global” group of stakeholders by conducting an internet–based survey of the affiliates to the “Global research priority setting network”, which had been assembled in the years prior to the development of the CHNRI method by the staff from the University of Toronto, Canada. Between March and May 2006 a large number of affiliates to the “Global research priority setting network” agreed to participate in a pilot on the condition of anonymity. They agreed to provide stakeholder input to five forthcoming exercises that aimed to set research priorities to address the five major causes of global child mortality. Respondents included a very diverse mix of researchers, policymakers and health practitioners with an interest in priority setting in health care from high–, middle– and low–income countries. Participants were given a simple version of the questionnaire, and were asked to rank the five “standard” CHNRI criteria from 1st to 5th in the order of their perceived importance of the criteria. They were also asked to set a threshold for each of the five criteria. The respondents placed the greatest weight (1.75) on potential for disease burden reduction, while the weights for the remaining four criteria were similar to each other, and ranged between 0.86 to 0.96. The highest threshold was placed on the criterion of answerability in an ethical way (0.54), while the lowest was placed on potential for disease burden reduction (0.39).

## CASE STUDIES OF STAKEHOLDER INVOLVEMENT IN CHNRI EXERCISES

We identified 50 research prioritization exercises using the CHNRI method that were published between 2007 and 2016. Of the 50 exercises, 38 (76%) did not seek inputs from stakeholders and 12 (24%) involved stakeholders as their larger reference group. This already shows how it may be remarkably difficult in most cases to identify and involve an appropriate group of stakeholders that would be representative of the wider community of interest – whether this is a global, regional, national or local population. It seems that, in the absence of simple solutions, most authors who conducted the CHNRI exercises preferred not to include stakeholders in the process, rather than including an ill–defined and non–representative group and then having to adjust the final ranks based on their input. By not including input from stakeholders, the CHNRI exercises simply remained “unfinished” to an extent, though weights and thresholds could still be applied *post–hoc* should an appropriate group of stakeholders be identified at some later stage – unless the context changes substantially in the meanwhile.

Among the 12 CHNRI exercises that involved stakeholders and took their input into account, 5 were papers that belonged to the series of exercises related to addressing research priorities for the five major causes of child mortality globally – eg, pneumonia, diarrhoea, neonatal infections, preterm birth/low birth weight, and birth asphyxia [7–11]. All of these papers were co–ordinated by the World Health Organization (WHO) and they used the weights and thresholds defined above by Kapiriri et al. [3]. However, the remaining seven exercises made their own individual attempts, using guidelines for implementation of the CHNRI method, to identify appropriate stakeholders within their own contexts and involve them in the process. This section explores the experiences and results from these seven studies. **Table 1** summarizes the approaches to involving stakeholders in these seven exercises.

**Table 1:** Summary tables on the involvement of stakeholders

Reference	Profiles and mode of identification	Number of stakeholders	Responsibility	Criteria	Weights and thresholds applied to the criteria	Impact of stakeholders' involvement on the final scores
[12]	Psychiatrists (9), psychologists (4), social workers (2), government employees (3), non–governmental organization representatives (6), researchers (6), users of mental health services (6) and members of the public service (7), including those from low–and middle–income countries; No indication as to how they were identified and selected	43	They were asked to rank the five pre–defined criteria with range of 1 to 5 (1–highest rank to 5–lowest rank)	5 standard CHNRI criteria used [4]	Weights were assigned based on ranking: effectiveness (+21%), maximum potential for burden reduction (+17%), deliverability (+0%), equity (–9%), answerability (–19%); Thresholds not applied	There was no description whether the ranks significantly differed between non–weighted and weighted scores
[13]	Mostly researchers and policy makers; also included technical experts, senior practitioners in the area of nutrition and child health (including 9 members of “MAMI” groups: Management of Acute Malnutrition for Infant less than six month reference group). Above profiles included all the participants and there was no clear description of the profile of stakeholders. Identified from the participants at meetings, symposia related to the technical area of concern	64	They were asked to score the research questions against the pre–defined criteria, rather than place weights on the criteria	5 standard CHNRI criteria (two composite criteria split into two – 7 in total) [4]	Weights and thresholds not applied	See main text: the stakeholder group was used for scoring, rather than weighting
[14]	Researchers, academics, clinicians, government officials, clinical psychologists, and member of the public. Identified based on their availability and accessibility with an attempt to ensure diversity of the group	30	Same as reference [12]	5 standard CHNRI criteria used [4]	Weights were defined using the rank given to the 5 pre–defined criteria: equity (+30%), efficacy and effectiveness (+9%), deliverability, affordability and sustainability (+2%), maximum potential for disease burden reduction (–9%), answerability and ethics (–19%); Thresholds not applied	The paper presented both the weighted and non–weighted scores. The stakeholders' inputs changed the ranking of the research options somewhat, but the top 20 research options remained the same in both cases
[15]	Scientists, students and lay people. Identified from staff members of the Public Health Foundation of India (PHFI) and those identified through personal networks of authors	Not mentioned	They are asked to rank the pre–defined five criteria from most important (ranked 1) to least important (ranked 5) within the national context	5 standard CHNRI criteria used [4]	Weights were defined using the rank given to five pre–defined criteria: deliverability, affordability (+18%), maximum potential for disease burden reduction (+18%), efficacy and effectiveness (+13%), equity (–17%) and answerability and ethics (–18%); thresholds not applied	The final outcome was not affected by the stakeholders' inputs on the criteria in that the top 15 research options remained the same across weighted and non–weighted scores
[16]	Managers from medical institutions, doctors, patients, and representatives of public (5 representatives of each group). Method of identification not mentioned	20	They were asked to rank the and also provide the thresholds on the pre–defined five criteria. However it was unclear whether or not other participants also provided the ranking to the criteria	5 criteria used: potential to affect change, maximum potential for disease burden reduction, deliverability, economic feasibility and equity	Weights: Potential to affect change (0.1925), maximum potential for disease burden reduction (0.1925), deliverability (0.2160), economic feasibility (0.1890) and equity (0.2050); Thresholds: Potential to affect change (33.5%), maximum potential for disease burden reduction (29.7%), deliverability (27.0%), economic feasibility (28.0%) and equity (27.8%).	It was unclear whether any major differences in the ranks were observed after applying the weights and thresholds
[17]	Obstetricians, gynaecologists, paediatricians, representatives of patients group, industry and international organizations; mode of identification was not mentioned	19	They were asked to rank the and also provide the thresholds on the pre–defined ten criteria	10 criteria used: answerability and ethics, efficacy and effectiveness, deliverability, maximum potential for disease burden reduction, equity, acceptability, sustainability, translation to policy, and economic feasibility and equity	Weights: answerability (0.11), efficacy and effectiveness (0.09), deliverability (0.10), maximum potential for disease burden reduction (0.14), equity (0.11) acceptability (0.07), sustainability (0.11), translation to policy (0.10), economic feasibility (0.10) and equity (0.07). Thresholds: answerability (33%), efficacy and effectiveness (38%), deliverability (28%), maximum potential for disease burden reduction (29%), equity (29%), acceptability (41%), sustainability (33%), translation to policy (33%), economic feasibility (40%) and equity (38%)	It was unclear whether any major differences in the ranks were observed after applying the weights and thresholds
[18]	The article addressed three country–led research prioritization exercises. In each country, stakeholders were researchers, academics, policy makers, district health workers, frontline health workers, implementing partners, people living with HIV/AIDS; mode of identification was not mentioned	40 to 70 participants each in Malawi, Nigeria and Zimbabwe	Stakeholders participated in the entire process ie, generation of research ideas and the scoring of research ideas. The weighting of scores was not applied in the exercise, because all stakeholders participated in the entire process.	6 criteria were used: answerability and ethics; potential maximum disease burden reduction on paediatric HIV infections; addresses main barriers to scaling–up; innovation and originality; equity; and likely value to policy makers	Weights and thresholds not applied	This exercise included diverse group of stakeholders. In this regard the relevance of the research ideas identified in the respective exercise to the national context was high.

Two exercises were carried out at the global level. They were focused on mental health research and acute malnutrition in infants less than six months, respectively [12,13]. The remaining five exercises were conducted at the national level and focused on research in child health in South Africa [14], zoonotic disease in India [15], health policy and maternal and child health in China [16,17], and Prevention of Mother–to–Child Transmission of HIV (PMTCT) in Malawi, Nigeria and Zimbabwe [18]. Given that the large majority (over 80%) of the 50 CHNRI exercises were focused on either the global context, or on all low– and middle–income countries (LMIC), the high representation of national–level exercises among those CHNRI studies that used stakeholders input (5/12) is likely a reflection of the fact that it is much easier to involve stakeholders at the national or sub–national level than it is on a regional or global level.

In all exercises, the stakeholders involved were first given an induction course about the CHNRI process. Then, an opportunity for asking and sharing questions and concerns with respect to the CHNRI process was provided. In five of the seven exercises, stakeholders were asked to rank the relative importance of the pre–defined criteria from most important one (“1”) to the least important (“5”), while considering the context of the research prioritization. The average score was calculated for each criterion and was then used to calculate the relative weights by dividing the average expected score of 3.0 (ie, the average expected rank if all criteria were valued the same) by the mean assigned rank. For example, a mean assigned rank for “answerability” criterion of 2.47 translates a relative weight of 1.21 (ie, 3.00/2.47 = 1.21). In this way, “answerability” will receive 21% greater weight than if all the criteria were weighted equally.

The concept of thresholds was very rarely used. Even when it was applied, it was clear that it wasn't properly explained to participating stakeholders. This is not surprising, because the thresholds really refer to a measure of “collective optimism” of the scorers, rather than a real computation of likelihood or probability that is rooted in any real–world parameters. It is very difficult to estimate what this measure of “collective optimism” could amount to for different criteria. This is why such attempts to set thresholds typically resulted in them being set at 25%–30%, much too low to have any discriminatory power and disqualify many research ideas, so that almost all research ideas passed all the thresholds.

In the remaining two exercises, the nature of stakeholder involvement was modified radically from that which was originally envisaged in the CHNRI exercises with reasonable justification [13,18]. Instead of using the group of stakeholders only to adjust the ranks that were derived from an expert–driven scoring process, the authors involved a broad range of stakeholders in the generation of research ideas [18] and/or scoring the research ideas [13,18]. We will now reflect on these experiences in a critical way, identify some lessons learnt and make recommendations for future exercises.

## CRITICAL ASSESSMENT OF STAKEHOLDER INVOLVEMENT IN CHNRI EXERCISES

In the 7 studies that tried to develop a larger reference group of stakeholders that would be appropriate to their respective contexts, the number of stakeholders involved was disappointingly small: it ranged from 20 to 70. Although attempts were clearly made to ensure diversity of the stakeholders involved, such small sample sizes can hardly be considered sufficiently inclusive of many different groups of stakeholders and their representativeness. Although good representativeness of stakeholders can be ensured without necessarily requiring a very large number of participants – such as, eg, in many examples of national parliaments in democratic societies, who represent all the people of the nation through a relatively small number of their elected members – we still feel that bigger numbers would ensure more legitimacy to the process, or more relevance of the outcomes to the context of the exercise.

**Figure:**
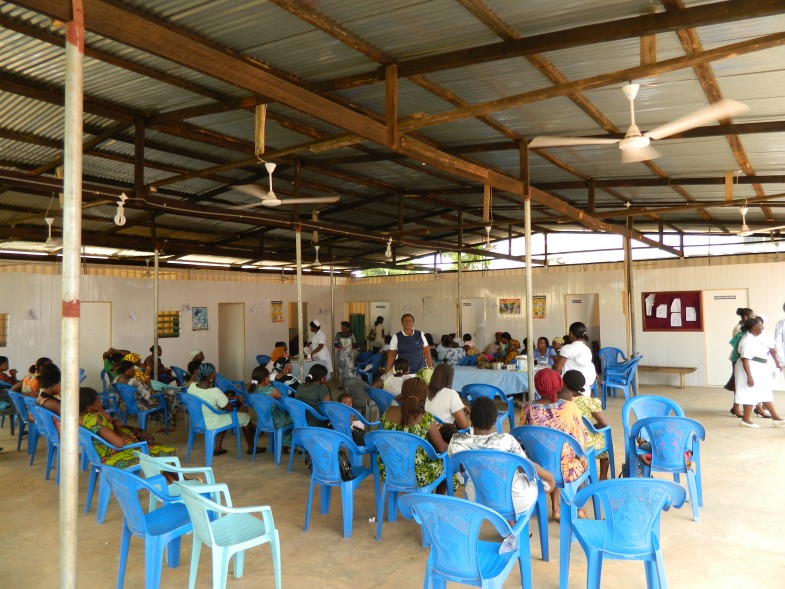
Photo: Meeting with a group of stakeholders at the maternity health clinic in Ghana (Courtesy of Dr Alice Graham, personal collection)

It would be difficult to consider the examples in the reviewed exercises as truly representative of the wider communities, let alone the nation or the world. This shows that despite the authors’ best intentions to fully adhere to the guidelines and complete the CHNRI process, they didn't really manage to find a satisfactory solution to involving large and diverse group of stakeholders. In these papers, the profile of stakeholders often included researchers, who would have been better reserved for the scoring process. Other stakeholders included clinicians, government officials, and representatives of academia and professional organizations, which again are rare in the society and hardly representative of the wider community. The examples of the profiles of persons who we would expect included in the larger reference group are also laypersons, frontline health workers and direct beneficiaries of health services, such as patients who contracted disease of concern. We encourage the authors of the future CHNRI exercises to try to get as much feedback as possible from those groups, because they have their own specialised knowledge (including lived experience), which would not be captured by other participating groups in the process. They also have “stake”, or interest, in the outcome of the exercise.

The small sample sizes and differences in approaches to ensure diversity and representativeness of the stakeholders led to large variations in stakeholders' input [12–18]. In the global exercise, the greatest relative importance was assigned to effectiveness, and the lowest to answerability, though these results should not be generalized. Stakeholders at the national level varied in their preferences, alternately supporting the criteria equity, deliverability (with affordability and sustainability), or the maximum potential for disease burden reduction (**Table 1**). Clearly, small sample sizes used in these exercises limit the generalizability of such preferences even within their local context, let alone more broadly.

It is also important to note that in all exercises that applied the “weights”, this procedure didn't really have dramatic effects on the final rankings of the research ideas. Although a research idea might move a few places up or down the list following the weighting procedure, these shifts did not profoundly affect the non–weighted ranking order that was determined by the researchers and experts. Perhaps this is one of the additional reasons why so many groups conducting the CHNRI exercise did not place sufficient importance on involving stakeholders. From the exercises that involved stakeholders, one might conclude that the process of expert scoring is sufficient and the outcome of the exercise will not be greatly altered by the involvement of stakeholders. We believe that such a view is premature and would like to see more examples of the involvement of the stakeholders in the CHNRI process before such judgements could be made.

In two exercises that actively involved stakeholders, their involvement wasn't limited to weights or thresholds, but rather they were also involved in research idea generation and scoring [13,18]. In the exercise on PMTCT in three African countries [18], about 40–70 people took part in respective countries, and all participants contributed to all stages of the CHNRI process. This included academics/researchers, district health workers and implementing partners such as UN agencies, people living with HIV/AIDS, frontline health workers and policy makers. The authors’ justification for including these diverse groups in all stages of the CHNRI process was to avoid discriminating within this diverse range of groups, but to truly engage the groups according to their technical expertise and to enhance inclusiveness and participation in similar priority–setting exercises across the nation. Eventually, the stakeholders' weighting of the scores was not even applied, possibly due to an assumption that it was no longer needed. This example represented a rather interesting deviation from the original CHNRI conceptual framework, but we can see a rationale for this modification, which makes it an illuminating exception.

The other exercise, on the management of acute malnutrition in infants in low– and middle–income countries, involved stakeholders only in the scoring process [13]. The stakeholder group included participants at meetings and symposia related to the topic area (**Table 1**). In this exercise, the core group of researchers (“management team”) developed the list of research questions based on the review of the literature in this field that preceded the CHNRI exercise as the preparatory step. The final list of questions was then circulated for scoring to both researchers invited to the CHNRI process and also the conference participants, who were considered stakeholders. Equal weighting was given to all criteria. The management team justified this on the grounds that malnutrition was a new area of research in infants younger than 6 months and they therefore believed that unweighted estimates would be more suitable and interpretable by their intended policy–maker audience. However, the authors stated that the lack of weighting of criteria might have resulted in limited reflection of the values in the broader community. In this case, we can conclude that the borderline between the invited researchers and the “stakeholders” (who were likely to include unrelated researchers and any other people of similar profile who could be expected to attend an international conference in this topic), was blurred and not really clear. It is likely that this deviation from the suggested approach didn't really invalidate the conceptual framework, because all the scorers would still be expected to possess knowledge on the topic of interest. It would perhaps be more appropriate not to call the second group “stakeholders”, but rather an additional, “convenience” sample of scorers that increased the number of scorers considerably.

## Proposed solutions and way forward

So far, there hasn't really been a good example of stakeholder involvement as originally envisaged by the CHNRI across the first 50 implementations, apart from perhaps the Kapiriri's priority–setting network involvement that was used in 5 child mortality papers [3,7–11]. This is certainly a shortcoming of all the previously conducted processes. This finding may also reinforce the initial concern that involving stakeholders in research priority setting processes is very challenging and that the solutions proposed in the original CHNRI method were quite difficult to implement as envisaged.

This is not to suggest that the results of previous CHNRI exercises are not useful, and the thresholds and weights can be applied later, if a good solution to obtain them can be found within the time scale during which the context described to scorers would still remain largely unchanged. The efforts conducted to date to perform the CHNRI exercises were not wasted and their results can be used. However, it must be acknowledged that most CHNRI exercises to date are, in fact, incomplete at least with respect to the original vision for them. To bridge this gap better definition is needed of who are the stakeholders at different levels (ie, global, regional, national and local) and how best to represent them.

For global exercises, we'll inevitably need a very large and inclusive crowd–sourcing exercise of many stakeholder representatives, who would place weights and thresholds on all 25 priority–setting criteria that were used to date across all 50 CHNRI exercises (5 “standard” and 20 new). The sample of stakeholders will need to be truly large, because we may later need several sub–samples that could provide us with region–specific stakeholders, or allow selecting specific groups of stakeholders and leaving others out of the exercise. In this way, the large “global” sample of stakeholders would also serve as a base for the regional samples of stakeholders. A major concern relating to this suggested approach would be how to avoid a strong urban bias in low–income settings and be inclusive of undeveloped and/or rural areas. In terms of national–level or local–level exercises, it is likely that highly targeted samples that aimed to include 500–1000 stakeholders would already be sufficient and representative of national or local context. The “targeting” component of the sampling strategy would define the profile of the stakeholders that would be most appropriate to the exercise, and then a person could be found in the community to fit each such profile.

How could these large sample sizes be achieved technically? How could we engage thousands of people globally, or hundreds nationally? With further attention to the development of the area of “crowd–sourcing” in the age of the internet and social networks (such as Facebook, Twitter, etc.), we should be able to do lot more in the future with respect to truly engaging the stakeholders in the process of setting priorities in health research investments at different levels of the human population.
